# How Useful Will Functional Proteomics Data Be?

**DOI:** 10.1002/cfg.101

**Published:** 2001-10

**Authors:** Pierre Legrain

**Affiliations:** Hybrigenics 3-5 Impasse Reille Paris 75014 France


**Comparative and Functional Genomics**
*Comp Funct Genom* 2001; **2**: 301–303.
**DOI:** 10.1002/cfg.101

**Conference Review**

### How useful will functional proteomics data be?

A presentation for the ESF workshop ‘Proteomics: Focus on protein interactions’

Pierre Legrain*
*Hybrigenics, 3-5 Impasse Reille, 75014 Paris, France*

**Correspondence to:
P. Legrain, Hybrigenics, 3-5
Impasse Reille, 75014 Paris,
France.
E-mail: plegrain@hybrigenics.fr*

Received: 22 July 2001
Accepted: 27 July 2001

**Keywords:** two-hybrid; bioinformatics; data set; protein interaction map; yeast; standards

### Introduction

Large-scale protein interaction maps are, with gene expression profiles, among the first examples of data sets generated without specific knowledge on functions of genes. These are technology-driven experiments rather than hypothesis-driven experiments. They are valuable tools for protein function prediction, despite the occurrence of typical artifacts. These approaches are still in their early stages. Related bioinformatics tools are also primitive and require much more independent experimental validation before becoming useful predictive tools. Functional annotations based on predictions cannot replace primary experimental sources of information that should also be accessible through functional databases available on the web. Ultimately, functional annotation asserting a putative function for a protein will certainly be replaced by a more precise description of experimental data on this protein, helping users of databases to build new hypotheses that still will have to be experimentally proven.

### Protein interaction maps

The yeast two-hybrid system [2] can detect interactions between two known proteins or polypeptides and can also search for unknown partners (prey) of a given protein (bait). Nevertheless, due to its intrinsic properties – that is measuring interactions between two chimeric and heterologous proteins in a yeast cell nucleus – a two-hybrid assay cannot apply to all protein-protein interactions and gives rise to a certain proportion of false positive and false negative results (for reviews see: [6,11]). During the last ten years, a few partial protein-protein interaction maps for viruses, bacteria and eukaryotes have been produced using different strategies, the matrix and the whole library approach. The matrix approach uses a collection of predefined open reading frames (ORFs), usually full-length proteins, as both bait and prey for interaction assays [4,12,13]. The library screening approach identifies for each interacting prey protein the domain of interaction with a given bait [3,9,13]. For both strategies, the rate of false positive interactions is difficult to evaluate and is largely dependent on the criteria applied for the significance of the interactions such as the reproducibility of results. Thus, to evaluate false positives and reproducibility, access to primary data is necessary. Datasets are becoming large, and specific bioinformatics tools should be designed to address these issues. Moreover, the two exhaustive studies of the yeast proteome done using the matrix approach have failed to recapitulate as much as 90% of interactions that were previously described in

Copyright © 2001 John Wiley & Sons, Ltd.

literature. This suggests a very high level of false negatives [4]. Thus, these datasets must be considered as largely incomplete and cannot sustain most statistical analyses that one would like to conduct on them. For example, Jeong and co-workers recently published an analysis of the public yeast protein interaction map [5]. The authors establish a positive correlation between connectivity and lethality: highly connected proteins are three times more likely to be essential. Although the existence of such a correlation makes biological sense, one should probably wonder about the relative weight of technological bias in establishing it. Jeong's work indeed relies mainly on interaction data produced by one systematic two-hybrid system in yeast. The corresponding protein interaction network, that gathers 1870 proteins (31% of the whole yeast proteome), is not complete. Its shape would probably be different if all 'real' interactions were known. Proteins that exhibit few interacting partners in this network could actually represent highly connected nodes. Conversely, false-positives of the two-hybrid system are likely to result in highly connected nodes of the network: so-called 'sticky prey' proteins bind 'by chance' to many independent bait proteins. The correlation between lethality and centrality in networks as evidenced by Jeong and co-workers, might actually be much stronger if genes that are both non-essential and highly connected on one hand, and genes that are both essential and poorly connected on the other hand, proved to be the consequences of a technological bias in data. In conclusion, global network analysis methods are fragile when poor quality or incomplete interaction data are used and the fact that the conclusion appears biologically sound (here an explanation of robustness) is not a proof of the validity of the demonstration *per se*.

Large proteomics projects demand resources seldom available to isolated academic or industrial laboratories. Distribution of work and pooling of resources within mixed academic/industrial networks will become increasingly necessary. Such collaborative efforts will revolve around the sharing of experimental data and post-analysis results. As yet nonexistent common databases will integrate data produced by several groups using different or even totally unrelated techniques. If these data are to be related meaningfully, the establishment of experimental standards for quality, of common computer representations for both experimental protocols and results, and of a set of corresponding benchmarks, are likely to become major issues within the proteomics community.

### Matching protein interaction maps with other data sets

Meaningful integration and useful presentation of linkage maps of the same biological nature obtained through diverse means is only the first step towards the fulfillment of proteomics' promises. Beyond 'single data-type' representation, analysis and visualization tools, one can envision more ambitious frameworks aimed at the comprehensive study of 'biological links' resulting from several distinct data types. Such compound generalized linkage maps should be fertile grounds for the discovery of more global insights into the molecular mechanisms of life. Efficient storage, manipulation and retrieval of experimental data, as well as the integration of technology-specific algorithms in a seamless chain of data transformation running from raw experimental data to exploitable results, are essential enablers of the scientific production process. This issue is now being raised for gene expression profiles [1]. The same will be needed for proteomics data.

Biologists (experts in a field) are the first users of proteomic and genomic data: they need exploration tools. An essential enabler for proteomics research should be the development of web-based graphical interfaces for the visualization, exploration and analysis of linkage maps resulting from each type of technology. As front-ends for databases following adequate standards of quality and presentation, these interfaces may provide not only a popular novel medium for the dissemination of scientific results, but also a mandatory complement to more traditional publishing. Bioinformaticians (experts in global analyses) are also users of the same data. Bioinformatic clustering of protein interactions represents a powerful annotation tool, which will become more and more useful as the interaction data accumulate. However, the two major hurdles for bioinformatic prediction algorithms are clearly the lack of independently validated methods, and the accuracy of functional genomics and proteomics datasets and annotations in databases [10]. Dealing with these issues means also solving the acute problem of naming genes and proteins in a unified way [8]. No integrated database will ever replace access to raw data for large-scale biology experiments and to the literature for description of

Copyright © 2001 John Wiley & Sons, Ltd.

functional assays, as rightly stated recently in an editorial in Science [7]. In order to be used successfully for appropriate functional annotation, the data needs to be stored in elaborate structures that allow each individual scientist to test his/her own hypothesis against complex heterogeneous primary data and then to design further experimental setting to validate the functional assignment.

### Acknowledgements

I thank Drs V. Schächter and J. Wojcik for very stimulating discussions.

### References

1. Davenport RJ. 2001. Microarrays – Data standards on the horizon. *Science* **292**: 414–415.
2. Fields S, Song O. 1989. A novel genetic system to detect protein–protein interactions. *Nature* **340**: 245–246.
3. Fromont-Racine M, Mayes AE, Brunet-Simon A, *et al.* 2000. Genome-wide protein interaction screens reveal functional networks involving Sm-like proteins. *Comp Funct Genom* **17**: 95–110.
4. Ito T, Chiba T, Ozawa R, Yoshida M, Hattori M, Sakaki Y. 2001. A comprehensive two-hybrid analysis to explore the yeast protein interactome. *Proc Natl Acad Sci U S A* **98**: 4569–4574.
5. Jeong H, Mason SP, Barabasi A-L, Oltvai ZN. 2001. Lethality and centrality in protein networks. *Nature* **411**: 41–42.
6. Legrain P, Wojcik J, Gauthier J-M. 2001. Protein–protein interaction maps: a lead toward cellular functions. *Trends in Genetics* **17**: 346–352.
7. Paydarfar D, Schwartz WJ. 2001. An algorithm for discovery. *Science* **292**: 13.
8. Pearson H, 2001. Biology's name game. *Nature* **411**: 631–632.
9. Rain JC, Selig L, de Reuse H, *et al.* 2001. The protein-protein interaction map of Helicobacter pylori. *Nature* **409**: 211–216.
10. Schwikowski B, Uetz P, Fields S. 2000. A network of protein-protein interactions in yeast. *Nat Biotechnol* **18**: 1257–1261.
11. Serebriiskii IG, Khazak V, Golemis EA. 2001. Redefinition of the yeast two-hybrid system in dialogue with changing priorities in biological research. *BioTechniques* **30**: 634–655.
12. Uetz P, Giot L, Cagney G, *et al.* 2000. A comprehensive analysis of protein–protein interactions in *Saccharomyces cerevisiae*. *Nature* **403**: 623–627.
13. Walhout AJ, Sordella R, Lu X, *et al.* 2000. Protein interaction mapping in *C. elegans* using proteins involved in vulval development. *Science* **287**: 116–122.

Copyright © 2001 John Wiley & Sons, Ltd. 
